# The human 2B4 and NTB-A receptors bind the influenza viral hemagglutinin and co-stimulate NK cell cytotoxicity

**DOI:** 10.18632/oncotarget.7597

**Published:** 2016-02-22

**Authors:** Alexandra Duev-Cohen, Yotam Bar-On, Ariella Glasner, Orit Berhani, Yael Ophir, Francesca Levi-Schaffer, Michal Mandelboim, Ofer Mandelboim

**Affiliations:** ^1^ The Lautenberg Center of General and Tumor Immunology, The Hebrew University Hadassah Medical School, IMRIC, Jerusalem, Israel; ^2^ Department of Pharmacology and Experimental Therapeutics, School of Pharmacy, Faculty of Medicine, The Hebrew University of Jerusalem, Jerusalem, Israel; ^3^ Central Virology Laboratory, Ministry of Health, Public Health Services, Chaim Sheba Medical Center, Ramat-Gan, Israel

**Keywords:** influenza, NK, 2B4, NTB-A, NKp46

## Abstract

Natural Killer (NK) cells are critical in the defense against viruses in general and against influenza in particular. We previously demonstrated that the activating NK cell receptor NKp46 is involved in the killing of influenza-virus infected cells through its interaction with viral hemagglutinin (HA). Furthermore, the recognition by NKp46 and consequent elimination of influenza infected cells were determined to be sialic-acid dependent. Here, we show that the human co-activating receptors 2B4 and NTB-A directly recognize the viral HA protein and co-stimulate killing by NK cells. We demonstrate that the 2B4/NTB-A-HA interactions require the sialylation of these receptors, and we identified the binding sites mediating these interactions. We also show that the virus counters these interactions through its neuraminidase (NA) protein. These results emphasize the critical role played by NK cells in eliminating influenza, a significant cause of worldwide morbidity and mortality.

## INTRODUCTION

NK cells are bone marrow derived lymphocytes that are part of the innate immune system [1]. They are able to kill virus-infected cells, bacteria, parasites and tumor cells [2]. Killing by NK cells is mediated by a balance between inhibitory and activating signals derived from inhibitory and activating receptors. This balance determines whether targets will be killed by NK cells or spared [3]. The inhibitory signals are primarily delivered upon binding of the NK inhibitory receptors to MHC class I proteins [4]. However, additional non-MHC class I binding receptors can also inhibit NK cell activity [5]. Three NK cell receptors: NKp30, NKp44 and NKp46, collectively named Natural Cytotoxicity Receptors (NCRs) are among the main activating NK receptors expressed by NK cells [2]. The NKp30 receptor recognizes tumor ligands such as B7H6 [6], and viral ligands such as pp65 [7] and the viral HA proteins of poxvirus and vaccinia virus [8]. The NKp46 receptor recognizes the HA proteins of influenza virus [9], poxvirus [8], sendai virus [9] and newcastle disease virus [10]. NKp46 also recognizes still unknown tumor [11] and cellular ligands [12, 13]. The NKp44 receptor binds to viral HA [14] and to tumor ligands, such as PCNA [15] and MLL5 [16].

Other receptors such as 2B4 and NTB-A function as co-receptors (i.e. the signal delivered from these receptors alone is not sufficient to induce killing by NK cells) [17]. The 2B4 and NTB-A receptors belong to the Signaling Lymphocyte Activating Molecule (SLAM) family [18]. 2B4 is expressed by all NK cells and by some T cell subsets [19]. Its high affinity ligand is CD48 [20]. NK-T-B Antigen (NTB-A) is expressed on all NK, T and B cells. It recognizes NTB-A by homophilic interactions [21].

Influenza virus infections annually result in worldwide health and economic burdens that affect the lives of millions [22]. Influenza is an RNA virus that encodes for 13 genes [23]. Two viral proteins: HA and neuraminidase (NA), are expressed on the viral envelope and on the infected cells’ membrane [24]. NK cells play an essential role in the recognition of influenza infected cells [11]. We previously showed that NKp44 and NKp46 bind to HA and this leads to NK-mediated killing of influenza virus infected cells [11]. We also showed that NK cells can eliminate the virus, via NKp44 and NKp46, even at the initial stages of infection when the virus only adheres to the cells [25]. The binding of NKp46 to the viral HA is dependent on the sialylation of the O-glycosylated residue, threonine 225, of NKp46 [26].

The mouse orthologous protein of NKp46 is named NCR1 [27]. Using mice deficient for NCR1 we demonstrated that NCR1 also directly interacts with viral HA and that the absence of NCR1 increases mice mortality due to influenza virus infection [25, 27]. Recently, we showed that the influenza virus counters the recognition of HA by NKp46 and NKp44 by using the viral NA, which cleaves sialic acids from these receptors [28, 29].

In the present study, we identified two NK cell receptors, 2B4 and NTB-A, that recognize HA in a sialic acid dependent manner. We show that the recognition of HA by 2B4 and NTB-A co-stimulates killing by NK cells. Furthermore, we were able to reveal the HA binding site on 2B4 and NTB-A.

## RESULTS

### Blocking of 2B4 and NTB-A reduces killing of influenza

**Figure 1:**
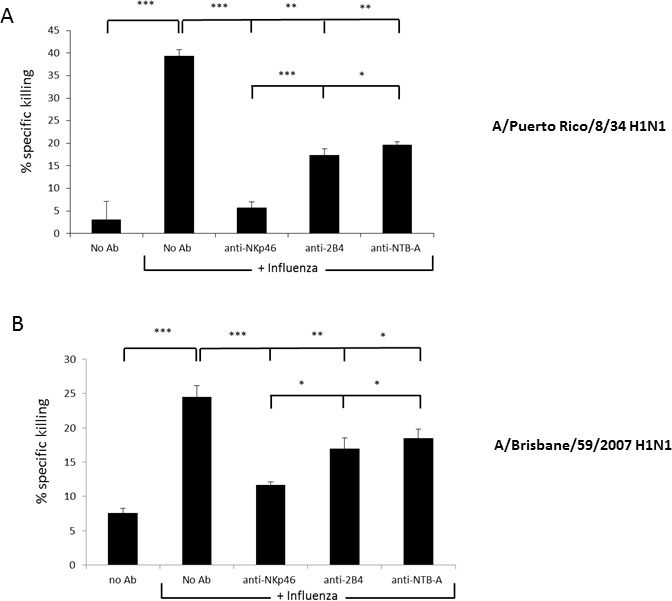
Blocking of 2B4 and NTB-A reduce killing of influenza JEG-3 cells were incubated with A/Puerto Rico/8/34 H1N1 **A.** or A/Brisbane/59/2007 H1N1 **B.** NK cells, which were pre-incubated with the polyclonal blocking antibodies indicated in the X axis, were then added to the Jeg3 cells and killing was determined 5 hours later. Shown are mean values and SD derived from triplicates, *p < 0.05, **p < 0.01, ***p < 0.005. Figure shows one representative experiment out of 3 performed.

**Figure 2:**
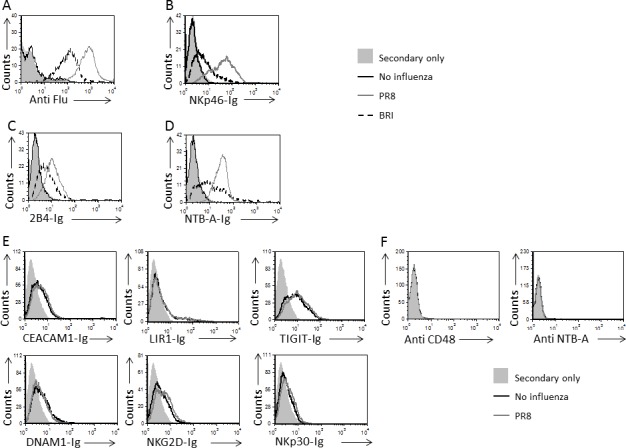
Enhanced binding of 2B4 and NTB-A to influenza **A.**-**D.** JEG-3 cells were incubated or not with A/Puerto Rico/8/34 H1N1 (PR8, gray empty histograms) or A/Brisbane/59/2007 H1N1 (BRI, dotted line histogram) viruses and then stained with anti-Flu mAb (A) and with the following fusion proteins (indicated on the X axis) NKp46-Ig (B), 2B4-Ig (C) and NTB-A-Ig (D). The black line empty histograms represent the staining of JEG-3 cells without influenza with anti-HA antibodies (A) or with the relevant fusion proteins (B-D) and gray filled histogram represents the staining of JEG-3 cells with secondary mAb only. For (A-D) the control staining of influenza-JEG-3 cells with secondary antibodies was similar to that of the wild type JEG-3 cells and is not shown in the figure. **E.** Staining of JEG-3 cells in the presence or absence of A/Puerto Rico/8/34 H1N1 with the proteins indicated on the X axis: CEACAM1-Ig, LIR1-Ig, TIGIT-Ig, DNAM1-Ig, NKG2D-Ig and NKp30-Ig. The black straight line empty histograms represent the staining of JEG-3 cells with the indicated fusion protein and the gray empty histograms represent the staining of the influenza-JEG-3 cells with the indicated fusion proteins. The filled histograms represent the staining of JEG-3 cells with secondary antibodies only. The control staining of influenza-JEG-3 cells with secondary antibodies was similar to that of the wild type JEG-3 cells and is not shown in the figure. **F.** Staining of JEG-3 cells in the presence or absence of A/Puerto Rico/8/34 H1N1 with the anti-CD48 and anti-NTB-A mAbs. The gray filled histogram represents the staining of JEG-3 cells with secondary mAb, the black line empty histograms represent the staining of JEG-3 cells without influenza and the gray empty histograms represent the staining of the influenza-JEG-3 cells. The control staining of influenza-JEG-3 cells with secondary antibodies was similar to that of the wild type JEG-3 cells and is not shown in the figure. Figure shows one representative experiment out of 3 performed.

We have previously shown that NK cells kill influenza infected cells through the interaction of NKp46 with the viral HA [9, 26, 28]. To test NK cell-mediated killing of cells coated with influenza, bulk primary human NK cell cultures were incubated with anti-NKp46 polyclonal antibodies and assayed against JEG-3 cells in the presence or absence of A/Puerto Rico/8/34 H1N1(PR8) (Figure 1A) or A/Brisbane/59/2007 H1N1 (BRI) (Figure 1B). We generated polyclonal antibodies by injecting the NKp46-Ig fusion protein into mice, 3 times, and collecting sera two weeks after the last injection was given. Specificity of the polyclonal antibodies was verified by FACS staining of cells which either express or do not express NKp46 (Figure S1). We used JEG-3 cells in these assays as they do not express any known activating NK ligands [29]. As can be seen, in the absence of influenza there was minimal killing of JEG-3 cells (Figure 1A and 1B). However, in the presence of both influenza strains, strong killing was observed (Figure 1A and 1B). As we previously reported [28], that this killing was dependent on NKp46, and indeed anti-NKp46 polyclonal antibodies successfully blocked the killing (Figure 1A and 1B). As controls, we used anti-2B4 and NTB-A polyclonal antibodies that were generated and verified their specificity (Figure S1). Surprisingly we observed a partial blocking of JEG-3 killing in the presence of PR8 and BRI viruses when either anti-2B4 or NTB-A polyclonal antibodies were included in the assays (Figure 1A and 1B, respectively). Because JEG-3 cells do not express CD48 (which is the 2B4 ligand) or NTB-A (which is the NTB-A ligand), prior to or following influenza infection (Figure 2), we hypothesized that both receptors might be involved in the killing of influenza and might recognize influenza virus proteins.

### 2B4 and NTB-A recognize the influenza virus

To test whether 2B4 and NTB-A recognize influenza virus directly, we generated fusion proteins composed of the extracellular portions of human 2B4 and NTB-A fused to the Fc portion of human IgG1 (named 2B4-Ig and NTB-A-Ig). Next, we incubated JEG-3 cells with influenza PR8 and BRI viruses and stained them with an anti-influenza antibody which recognizes components of both viruses (Figure 2A). We used this mAb, since we want to see whether there is a correlation between the levels of influenza infection and recognition by NKp46-Ig, that was used as a positive control, 2B4-Ig, and NTB-A-Ig. As previously reported [28], enhanced binding of NKp46-Ig was observed to the PR8/BRI-JEG-3 cells when compared to non-infected cells (Figure 2B). Importantly, increased binding of 2B4-Ig (Figure 2C) and NTB-A-Ig (Figure 2D) to the PR8/BRI-JEG-3 cells was also observed. In all cases, the binding was correlate with the levels of infection (Figure 2A), and binding to PR8-coated cells was better than BRI (Figure 2B-2D).

The enhanced binding of 2B4 and NTB-A was specific, as no additional NK cell receptor fusion proteins bound the PR8 infected cells (Figure 2E). Similar results were obtained with BRI-JEG-3 cells (data not shown). CD48 and NTB-A (the 2B4 and NTB-A cellular ligands, respectively) were not expressed on JEG-3 cells either prior to infection or following infection with PR8 (Figure 2F), or with BRI (data not shown).

### 2B4 and NTB-A bind viral HA in a sialic acid-dependent manner

**Figure 3:**
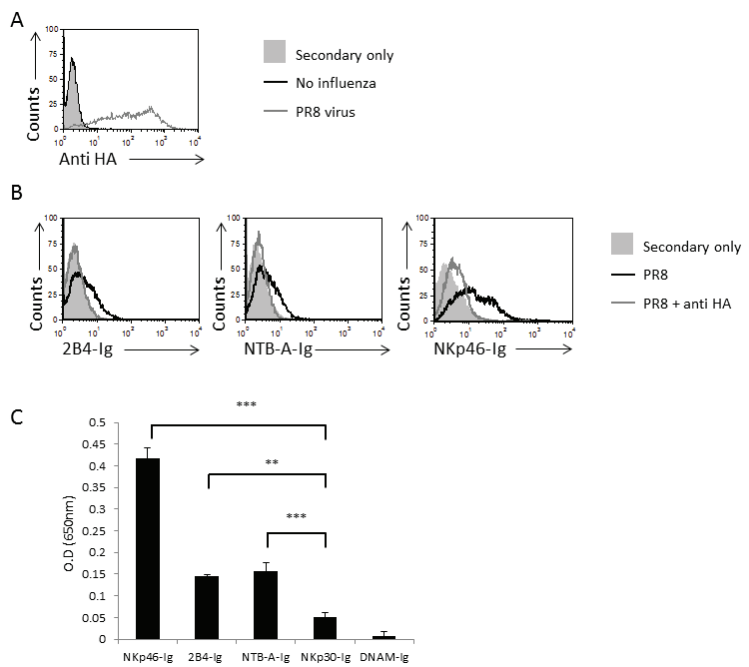
2B4 and NTB-A directly interacts with viral HA **A.** JEG-3 cells were incubated or not with PR8 influenza virus and stained with anti-HA mAb. The black empty histogram represents the staining of JEG-3 cells. The gray empty histograms represent the staining of JEG-3 cells in the presence of influenza. The filled histograms represent the staining of JEG-3 cells with secondary mAb only. The background staining of the JEG-3 cells in the presence of influenza with secondary antibodies was similar to that of the JEG-3 cells and is therefore not shown in the figure. **B.** JEG-3 cells were incubated with influenza A/Puerto Rico/8/34 H1N1 and then incubated with or without anti-HA antibody, followed by staining with 2B4-Ig, NTB-A-Ig and NKp46-Ig (indicated on the x axis of the histograms). The filled histograms represent the staining of JEG-3 cells with the various fusion proteins, the black empty histograms represent the staining of the influenza-JEG-3 cells and the gray empty histograms represent the staining of the influenza-JEG-3 cells with the indicated fusion proteins, following blocking of the HA. Figure shows one representative experiment out of 3 performed. **C.** Various fusion proteins (indicated on the X axis) were coated on an ELISA plate and then biotinylated HA-Ig was added. Shown are mean values and SD derived from triplicates, **p < 0.01, ***p < 0.005. Figure shows one representative experiment out of 3 performed.

We previously demonstrated that NKp46 interacts directly with viral HAs and that this interaction is sialic acid-dependent [9]. To investigate whether 2B4 and NTB-A also bind viral HA, we incubated the JEG-3 cells with influenza PR8. The virus adheres to the cells as seen by staining with an anti-HA1 antibody (Figure 3A). We then blocked the viral HA protein on the influenza-JEG-3 cells with an anti-HA blocking mAb and stained the blocked and unblocked cells with 2B4-Ig, NTB-A-Ig and NKp46-Ig (used as a positive control). As can be seen in Figure 3B, the binding of all 3 fusion proteins to influenza-coated cells was nearly abolished following the blocking of HA, indicating that 2B4 and NTB-A interact directly with HA, similarly to NKp46.

To further demonstrate that 2B4, NTB-A and NKp46 bind directly to HA we performed ELISA assays in which we coated ELISA plates with the different fusion proteins and tested the binding to biotinylated HA-Ig. A significant binding of HA to NKp46-Ig was detected and a moderate binding to 2B4-Ig and NTB-A-Ig, (Figure 3C).

**Figure 4:**
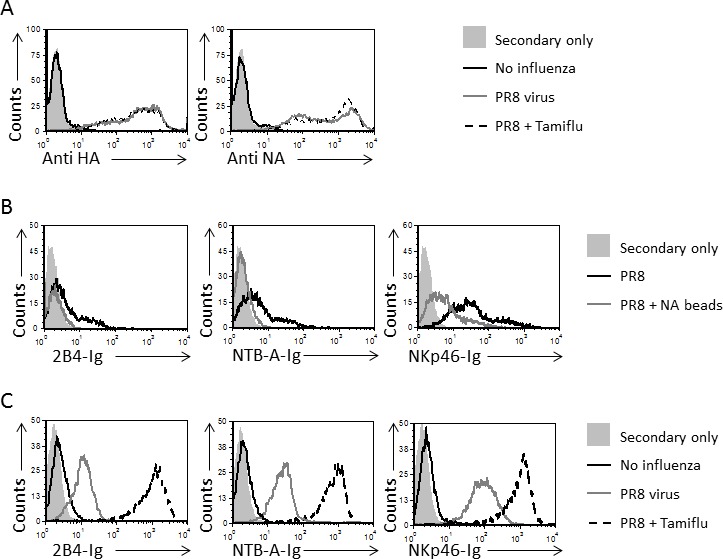
The binding of 2B4 and NTB-A to HA is sialic acid dependent **A.** JEG-3 cells were incubated or not with influenza and then stained with anti-HA and anti-NA mAbs (indicated on the X axis) before (gray empty histograms) and after Tamiflu treatment (dotted line histograms). The black empty histograms represent staining of JEG-3 influenza cells with the appropriate mAbs and the filled gray histograms represent the staining of the WT JEG-3 cells with the secondary antibodies only. The background staining of the JEG-3 cells in the presence of Tamiflu with secondary antibodies was similar to that of the JEG-3 cells in the absence of Tamiflu and is therefore not shown in the figure. **B.** JEG-3 cells were incubated with influenza and then stained with fusion proteins (indicated on the x axis) that were treated (gray line histograms) or not (black line histograms) with NA prior to the staining. The filled histograms represent the staining of the WT JEG-3 cells with the secondary antibodies only. **C.** JEG-3 cells were incubated or not with influenza and then treated or not with Tamiflu. Cells were then stained with the fusion proteins that are indicated on the X axis. Staining of JEG-3 cells is indicated by the black empty histograms, staining of influenza-JEG-3 cells is indicated by the gray empty histograms and staining of the influenza-JEG-3-Tamiflu treated cells is indicated by the dotted line histograms. The filled histograms represent the staining of WT JEG-3 cells with the secondary antibodies only. The background staining of the influenza-JEG-3 cells in the presence or absence of Tamiflu with secondary antibodies was similar to that of the WT JEG-3 cells and is therefore not shown in the figure. Figure shows one representative experiment out of 3 performed.

To test whether sialylation of 2B4 and NTB-A is required for their binding to HA, as described for NKp46 [28], we treated NKp46-Ig, 2B4-Ig and NTB-A-Ig with NA beads to remove sialic acids from these receptors. The treated fusion proteins were subsequently used for staining of the influenza-coated JEG-3 cells (Figure 4A shows staining of the influenza-JEG-3 cells by anti-HA and anti-NA mAbs). A significant decrease in the binding of 2B4-Ig and NTB-A-Ig, as well as NKp46-Ig, was observed following treatment of the fusion proteins with NA (Figure 4B), indicating that interaction of NKp46, 2B4 and NTB-A, with HA, is sialic acid-dependent.

The NA protein is expressed on influenza-infected cells and on the virus itself (Figure 4A and [33]). We previously demonstrated that NA counters the recognition of NKp46 via the cleavage of sialic acids from NKp46 and that blocking of the NA activity results in enhanced NKp46 recognition of HA [28]. To test whether blocking of NA activity will result in increased 2B4 and NTB-A recognition, we incubated the influenza-JEG-3 cells with oseltamivir carboxylate (Tamiflu), a drug that blocks NA activity [28], and then incubated the cells with the fusion proteins. The Tamiflu treatment did not alter the expression of HA and NA on the coated-influenza cells (Figure 4A). A substantial increase in the binding of 2B4-Ig, NTB-A-Ig and NKp46-Ig was observed (Figure 4C), indicating that NA antagonizes the binding of all of these receptors. Collectively, these findings indicate that 2B4 and NTB-A, similarly to NKp46, directly recognize HA in a sialic acid-dependent manner and that NA antagonizes these interactions.

### 2B4 and NTB-A are activated following influenza recognition

**Figure 5:**
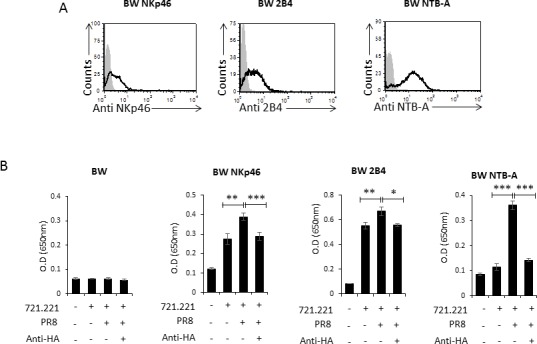
The 2B4 and NTB-A receptors are co-stimulated following interaction with influenza **A.** BW cells were transfected to express the NKp46, 2B4 and NTB-A receptors (BW NKp46, BW 2B4 and BW NTB-A, respectively). Shown are the transfectant cells stained with anti-NKp46 (left histogram), anti-2B4 (middle histogram) and anti-NTB-A (right histogram). The filled gray histogram represents the staining with the secondary antibodies only. Figure shows one representative staining out of 3 performed. **B.** 721.221 cells were incubated or not (indicated by + and −) with influenza and with or without anti HA blocking antibody, followed by incubation with the various BW cells: BW, BW NKp46, BW 2B4 and BW NTB-A (indicated above the graphs). IL-2 in the supernatants was determined after 48 hours of incubation by using ELISA. Shown are mean values and SD derived from triplicates, *p < 0.05, **p < 0.01, ***p < 0.005. Figure shows one representative experiment out of 3 performed.

To test whether 2B4 and NTB-A can be directly activated by influenza virus we used a cell based reporter system. In this system, we expressed chimeric proteins composed of the extracellular portions of NKp46, 2B4 and NTB-A fused to the mouse ζ-chain in mouse BW cells. The BW cells secrete IL-2 upon engagement of the chimeric protein with an appropriate ligand [30], thus reporting not only on the binding of a particular ligand but also its functionality. After verifying the expression of NKp46, 2B4 and NTB-A chimeras on the BW cells (Figure 5A), we incubated the various BW cells with 721.221 and with PR8 influenza-coated 721.221 cells. Following a 48 hour incubation, we determined the amount of IL-2 in the culture supernatants by ELISA. 721.221 cells (with and without influenza) were used as targets since they express an unknown tumor ligand for NKp46 [9] and also cellular ligands for NTB-A (NTB-A) and 2B4 (CD48) [31, 32]. In addition, we incubated the PR8 influenza-coated cells with the anti HA blocking antibody. When the parental BW cells were incubated with 721.221 there was no secretion of IL-2 (Figure 5B). In contrast, a significant increase in IL-2 levels was observed in the culture supernatants when all 3 transfectant cells: BW NKp46, BW 2B4 and BW NTB-A were incubated with 721.221 cells (Figure 5B), indicating that the reporter system functions and that upon interaction of the various chimeric reporter proteins with their cellular ligands, IL-2 is secreted. Importantly, IL-2 secretion from the BW NKp46, BW 2B4 and BW NTB-A cells was further enhanced following incubation of the 3 reporter BW cells with PR8 influenza-721.221 (Figure 5B), indicating that the 3 receptors directly recognize influenza-coated cells. Moreover, when the influenza coated cells were incubated with anti HA blocking antibody there a reduction in IL-2 secretion was detected and IL-2 levels were similar to those observed without influenza (Figure 5B). Similar results were obtained with the BRI strain of influenza (Data not shown).

### Identification of the 2B4 and NTB-A-HA binding site

**Figure 6:**
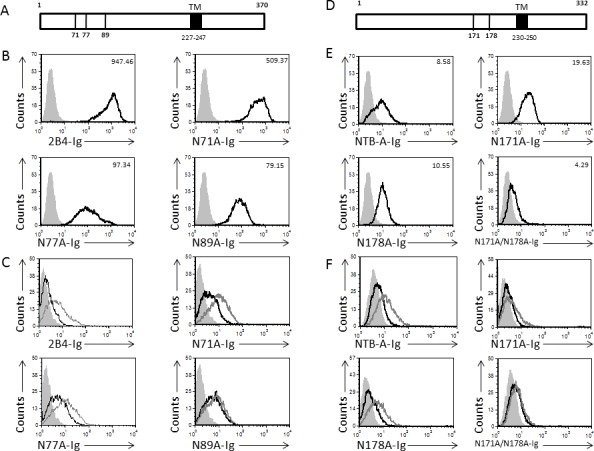
Identification of 2B4 and NTB-A binding site **A.**, **D.** Schematic representation of the 2B4 (A) and the NTB-A (D) human receptors. The location of the N-linked glycosylation sites are indicated (71, 77 and 89 for 2B4 (A) and 171, 178 for NTB-A (D)). The transmembrane region is indicated in black and its amino acid position in the protein is indicated by numbers. **B.**, **E.** FACS staining of 721.221 cells performed with wild type 2B4-Ig and with the various 2B4-Ig mutated proteins (indicated on the X axis (B)) and with the NTB-A-Ig and the various NTB-A mutants (indicated in the X axis (E)). The filled histogram represents the staining with secondary antibodies only. Figure shows one representative experiment out of 3 performed. **C.**, **F.** FACS staining of JEG-3 cells with and without influenza performed with the wild type 2B4-Ig and with the various 2B4-Ig mutated proteins (indicated on the X axis (C)) and with the NTB-A-Ig and the various NTB-A mutants (indicated on the X axis (F)). The black empty histogram represents the staining of JEG-3 cells and the gray empty histogram represents the staining of the JEG-3 cells in the presence of influenza. The filled histogram represents the staining of the JEG-3 cells with secondary antibodies only. The staining of the JEG-3 cells in the presence of influenza with the secondary antibodies only was similar to the JEG-3 cells and is therefore not shown in the figure.

To identify the NTB-A and 2B4 binding site that mediates their interaction with HA, we focused on the glycosylated residues of these two receptors, because we demonstrated that glycosylation is essential for their binding. The 2B4 receptor contains three N- glycosylated residues (Asn71, Asn77 and Asn89, Figure 6A and [34]). To identify which of these residues were critical for the 2B4 recognition of HA, we introduced point mutations in each of them, converting them to alanine. We then cloned the extracellular portions of each mutated protein in frame with the Fc portion of human IgG1 and produced fusion proteins.

Initially, we stained the 721.221 cells to investigate whether the sugar carrying residues of 2B4 play a role in the recognition of its cellular ligand CD48. In agreement with previous reports [34], we observed that all 2B4 sugar-carrying residues (in particular N77 and N89) play a role in CD48 recognition, as mutating each of the three residues reduced the binding of 2B4 to CD48. The MFI was reduced significantly as indicated in the histogram (Figure 6B). Next, we tested the binding of all 2B4 mutated proteins to influenza-JEG-3 cells. As can be seen, for HA binding to 2B4, N89 is the critical residue since mutating it completely abolished the increased binding observed following target cell incubation with influenza (Figure 6C).

The NTB-A receptor was predicted to have two N glycosylated residues at positions Asn171 and Asn178 [35]. To test which of these residues mediates the binding of NTB-A to HA, we initially mutated each of these residues to alanine and produced the corresponding fusion proteins N171A-Ig and N178A-Ig. We next used these fusion proteins to stain 721.221 cells (to test whether the mutations will affect their binding to their cellular ligand NTB-A), and to stain JEG-3 cells in the absence or presence of influenza (to test whether the mutations will affect NTB-A-HA interactions). The single mutations had little effect on NTB-A homotypic interactions (Figure 6E) and binding to HA was also minimally affected (Figure 6F). We therefore generated another fusion protein, in which both glycosylated residues were mutated, and denoted it N171A/N178A-Ig. Mutating both glycosylated residues only slightly reduced binding to NTB-A (Figure 6F). However, the binding to HA was completely abolished (Figure 6F). These findings indicate that Asn89 of 2B4 mediates the binding of 2B4 to viral HA, and that both Asn171 and Asn178 together are critical for the binding of NTB-A to viral HA.

### The mouse 2B4 and mouse NTB-A do not bind HA

**Figure 7:**
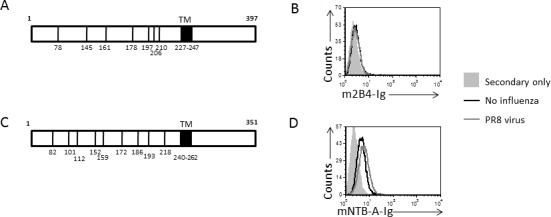
The mouse 2B4 and NTB-A receptors do not bind to influenza **A.**, **C.** Schematic representation of the mouse 2B4 (m2B4, (A)) and the mouse NTB-A (mNTB-A, (C)) receptors. The location of the N-linked glycosylation sites are indicated (78, 145, 161, 178, 197, 206 and 210 for 2B4 (A) and 82, 101, 112, 152, 159, 172, 186, 193 and 218 for NTB-A (C)). The transmembrane region is indicated in black and its position in the protein is indicated by the amino acids numbers. **B.**, **D.** FACS staining of JEG-3 cells in the presence and absence of influenza with m2B4-Ig (B) and the mNTB-A-Ig (D) fusion proteins. The black empty histogram represents the staining of JEG-3 cells, the gray empty histogram represents the staining of JEG-3 cells in the presence of influenza and the filled histogram represents the staining of the JEG-3 cells with secondary antibodies only. The staining of the JEG-3 cells in the presence of influenza with the secondary antibodies only was similar to the JEG-3 cells and is therefore not shown in the figure. Figure shows one representative experiment out of 3 performed.

The mouse homologue of NKp46, named NCR1, was reported to bind HA [25]. We therefore investigated whether the mouse 2B4 (m2B4) and mouse NTB-A (mNTB-A) receptors would also interact with HA. The glycosylation pattern of m2B4 is substantially different from that of human 2B4 (compare Figures 7A and 6A) and residue Asn89 which mediates the 2B4-HA interactions is absent from m2B4. Accordingly, m2B4 did not demonstrate increased binding to influenza-JEG-3 cells (Figure 7B). Similarly, the glycosylation pattern of mNTB-A is also different from the human NTB-A, and both Asn171 and Asn178 residues which are critical for binding of human NTB-A to viral HA are absent in the mouse (compare Figures 7C and 6D). Indeed, no increased binding of mNTB-A-Ig was observed to influenza-JEG-3 cells (Figure 7D).

## DISCUSSION

Four documented influenza pandemics occurred in the last two centuries and annual influenza infections constitute a major health and economic problem [36]. Therefore, it is essential to understand in detail how immune cells respond to influenza virus infection. To avoid recognition by the immune system, influenza viruses undergo rapid changes [36]. In particular, the two major envelope proteins of influenza: HA and NA which are also expressed on infected cells’ membrane, are subject to intense changes in their amino acids sequences [36]. However, despite these changes, the function of these two proteins is not altered; i.e practically, all HA proteins will bind sialic acids and all NA proteins will cleave sialic acids.

Approximately four decades ago it was demonstrated that NK cells are critical for fighting influenza infections [37]. However, the molecular mechanisms by which NK cells recognize influenza were unknown, and it was unclear how NK cells can recognize various strains of influenza viruses. We have shown that NK cells recognize influenza viruses primarily through the interaction of NKp46, but also of NKp44, with the viral HA protein [14]. We further demonstrated that NKp46 utilizes the conserved ability of HA to bind sialic acids in order to interact with HA, as sialylation of NKp46 is required for these interactions. The virus, however, fights back by using its NA protein to counteract the interaction of NKp46 and HA by removing the sialic acids from NKp46 [28].

We began this research after observing decreased killing of two influenza virus strains by NK cells after blocking of the 2B4 and NTB-A receptors. Using Ig fusion proteins we verified that both the 2B4 and NTB-A receptors directly interact with influenza virus. We further demonstrated that 2B4/NTB-A bind to HA and that the interaction is similar to that of NKp46. It is sialic acid dependent and NA antagonizes these interactions.

Interestingly, the presence of sialic acids *per se* is not sufficient for binding to HA as other NK cell receptors were unable to interact with HA although all of them are sialylated. Thus, there are elements other than the presence of sialic acids which determine the specificity of NK receptor-HA interactions.

In primary NK cells, both 2B4 and NTB-A function as co-stimulatory receptors [17]. Indeed, when we blocked 2B4 and NTB-A, the increased killing of influenza was partially reduced. However, it was completely abrogated when NKp46 was blocked. This indicates that NKp46 (and also NKp44) mediates the killing of influenza by binding to HA, and that 2B4 and NTB-A co-stimulate this killing.

We also determined the 2B4 and NTB-A binding sites to HA (asparagine 89 in 2B4 and asparagine 171 and 178 in NTB-A). We further demonstrated (as previously reported) that these glycosylated residues are also involved in the binding of 2B4 and NTB-A to their corresponding cellular ligands: CD48 and NTB-A, respectively. Interestingly, in NKp46 an O-glycosylated residue (Threonine 225) mediates the activating interaction with HA, while in 2B4 and NTB-A N-glycosylated residues mediate co-stimulatory interactions with HA.

We previously showed that the mouse NKp46 orthologous protein named NCR1 directly interacts with HA, and that increased mortality of mice infected with influenza virus is observed in the absence of NCR1 [25]. Conversely, the m2B4 and the mNTB-A did not interact with HA. Indeed, the amino acids of human 2B4 and NTB-A that are required for their binding to HA are not conserved in the mouse. In addition the m2B4, as opposed to the human 2B4, sometimes function as an inhibitory receptor [38]. Interestingly, we recently discovered that Thr 225 is also O-glycosylated in the mouse NCR1 and that this residue (similarly to NKp46) mediates the interaction between Ncr1 and HA (Glasner et al., in press).

The identification of two additional NK cell receptors that directly interact with viral HA and the understanding that NA counters the interactions of these two co-stimulating receptors with HA might lead to the development of new therapeutic approaches against this dangerous virus that are based on either blocking the NA activity or on augmenting the sialylations of NKp46, NKp44, 2B4 and NTB-A receptors.

## MATERIALS AND METHODS

### Cells and viruses

The cell lines used in this study were the human choriocarcinoma cell line JEG-3, the EBV transformed B cell line 721.221, and the murine thymoma BW cell line. The human influenza viruses A/Puerto Rico/8/34 H1N1 and A/Brisbane/59/2007 H1N1 used in this study were generated as previously described [30]. NK cells were isolated from peripheral blood lymphocytes using the Easy Sep Negative selection human NK cells enrichment kit purchased from Stemcell Technologies according to the manufacturer's instructions.

### Antibodies, fusion proteins and compounds

Monoclonal antibodies (mAbs) used in the present study included anti-influenza type A mAb (anti Flu) (Center for Disease Control Atlanta Georgia) that recognizes components of Influenza A viruses, anti-HA1 mAb (H17-L2) that recognizes the HA protein of A/Puerto Rico/8/34 H1N1, anti-NA1 (NA21C1) and the blocking antibody anti-HA1 (H28E23) (all are kind gifts from Jonathan Yewdell, National Institute of Health). The anti-2B4 (c1.7), anti-NTB-A (NT-7) and anti-NKp46 (9E2) mAbs were purchased from Biolegend. Anti-CD48 (eBio156-4H9) was purchased from eBioscience. The polyclonal anti-2B4, anti-NTB-A and anti-NKp46 were generated by immunization of male C57BL mice with 2B4-Ig, NTB-A-Ig and NKp46-Ig, respectively. The specificity of these polyclonal antibodies was tested against transfectants expressing the appropriate receptors. PE-conjugated AffiniPure donkey anti-human IgG and Alexa Fluor 647-conjugated AffiniPure goat anti-mouse IgG were purchased from Jackson ImmunoResearch. The generation of the following proteins: NKp46-Ig, LIR1-Ig, NKp30-Ig, CEACAM1-Ig, DNAM1-Ig, TIGIT-Ig, NKG2D-Ig, HA-Ig was previously described [26]. All proteins were generated in 293T cells and were purified on a protein G column as previously described [26]. The purity of all fusion proteins used in this work was near 100%. HA-Ig used in the ELISA experiment was biotinylated (Thermoscientific, #21331). For neuraminidase (NA) inhibition, Tamiflu (Oseltamivir carboxylate (Santa cruz, sc-212484) was used. Treatment of the fusion proteins with NA beads (Sigma) was performed as previously described [26].

### Generation of 2B4-Ig, NTB-A-Ig, mouse 2B4-Ig, mouse NTB-A-Ig and mutations in 2B4-Ig and NTB-A-Ig

For the generation of the WT 2B4-Ig fusion protein, the sequence encoding the extracellular part of 2B4 was amplified by PCR using the 5′ primer CCCACCGGT GCCGCCACC ATG CTG GGG CAA GTG GTC ACC (including AgeI restriction site) and the 3′ primer GGATCCGG CCA AAA TCT GAA TTC CTG (including BamHI restriction site). Single point mutations in 2B4 were generated by PCR- based site directed mutagenesis using the 5′ primer AAG TGG GAG GCT GGC TCT TTG and the 3′ primer CAA AGA GCC AGC CTC CCA CTT for N71A mutation, the 5′ primer TTG CCT TCC GCT ACT TCC AAT and the 3′ primer ATT GGA AGT AGC GGA AGG CAA for the N77A mutation, the 5′ primer ATA GTC AAG GCC TTG AGT CTTC and the 3′ primer GAA GAC TCA AGG CCT TGA CTAT for the N89A mutation. For the generation of the WT NTB-A-Ig the 5′ primer CCC ACCGGT GCCGCCACC ATG TTG TGG CTG TTC CAA TCG (Including AgeI restriction site) and the 3′ primer GGG ACTCAT TTT GGT ATC TGT ATA TTG (Including BamHI restriction site) were used. The single point mutations of NTB-A were generated by PCR based site directed mutagenesis using the 5′ primer GCC TTG GGA GCC ACA CTT TCA and the 3′ primer TGA AAG TGT GGC TCC CAA GGC for the N171A mutation, the 5′ primer AGT CAG CCA GCC CTC ACT GT and the 3′ primer AC AGT GAG GGC TGG CTG ACT for the N178A mutation. The N171A PCR fragment was used with the N178A primers to generate a double mutated PCR fragment. These PCR fragments were cloned into an expression vector containing a mutated Fc portion of human IgG1 (CSI-Ig Puro plasmid). For the generation of the mouse 2B4-Ig fusion protein, the sequence encoding the extracellular part of mouse 2B4 was amplified by PCR using the 5′ primer CCC ACCGCT GCCGCCACC ATG TTG GGG CAA GCT GTC CTG (including AgeI restriction site) and the 3′ primer GGG ATCCGG CAG AAA TCT GAA ATT CGAA (including BamHI restriction site). For the generation of the mouse NTB-A-Ig fusion protein, the sequence encoding the extracellular part of mouse NTB-A was amplified by PCR using the 5′ primer CCCACCGGT GCCGCCACC ATG GCT GTC TCA AGG GCT CAA (including AgeI restriction site) and the 3′ primer GGGATCCCA GGG TGG ATT AGT TAG AAC (including BamHI restriction site).

### FACS staining and viral coating

Cells were coated overnight with 50μl of the A/Puerto Rico/8/34 H1N1 virus strain at 37°C. The cells were then washed, incubated with the appropriate antibody (0.2μg/well) or fusion protein (1μg to 5μg/well), stained with the appropriate secondary antibody and then analyzed by fluorescence-activated cell sorting (FACS). For NA inhibition or HA blocking, the cells were incubated for 1h on ice with Tamiflu (1:1000) or anti-HA (H28E23 0.5μg/well)) and then stained with the appropriate fusion protein. All results were analyzed with the CellQuest software.

### Killing assay

NK cells were incubated for 5 hours at 37°C with various targets and the cytotoxic activity was measured by ^35^S release as described previously [31]. For blocking of 2B4, NTB-A and NKp46 receptors, NK cells were incubated with the appropriate polyclonal antibody (1:1000, prepared as described above) for 1h on ice prior to co-incubation with the target cells.

### BW assay

The cloning of chimeric 2B4- ζ chain constructs was performed by PCR amplification. The primers used for the extracellular portion of 2B4 were: The 5′ primer CCC AAGCTT GCCGCCACC ATG CTG GGG CAA GTG GTC AC (Including HindIII restriction site) and the 3′ primer TCC ATC TAG CAA GTA GCA GAG CGG CCA AAA TCT GAA TTC CTG A (including the seven first amino acids of the mouse ζ-chain transmembrane portion). The mouse ζ-chain was amplified by using the 5′ primer CAG GAA TTC AGA TTT TGG CCG CTC TGC TAC TTG CTA GAT GGA A (including the last seven amino acids of the human 2B4 extracellular portion) and the 3′ primer GGC TCG AGT TAG CGA GGC C AGGGTC (Including XhoI restriction site). The two amplified fragments were mixed and PCR was performed for the generation of 2B4- ζ-chain construct. The cloning of chimeric NTB-A- ζ chain constructs was performed by PCR amplification. The primers used for the extracellular portion of NTB-A were: The 5′ primer C GAATTC GCCGCCACC ATG TTG TTG CTG TTC CAA TCG (Including EcoRI restriction site) and the 3′ primer TCC ATC TAG CAA GTA GCA GAG CAT TTT GGT ATC TGT ATA TTG AATT (including the seven first amino acids of the mouse ζ-chain transmembrane portion). The mouse ζ-chain was amplified by using the 5′ primer CAA TAT ACA GAT ACC AAA ATG CTC TGC TAC TTG CTA GAT GGA A (including the last seven amino acids of the human 2B4 extracellular portion) and the 3′ primer GGC TCG AGT TAG CGA GGC C AGGGTC (Including XhoI restriction site). The two amplified fragments were mixed and PCR was performed for the generation of NTB-A- ζ-chain construct. The constructs (2B4- ζ-chain and NTB-A- ζ-chain) were cloned into a pcDNA3 expression vector and transfected into BW cells. BW-NKp46 cells were previously generated in our lab [9].

BW assays were performed as previously described [30]. Parental BW or BW transfected cells were incubated with irradiated targets (6000rad) at 1:2 effector to target (E:T) ratio. After 48h, the supernatants were collected and levels of interleukin-2 (IL-2) were quantified by sandwich ELISA using anti IL-2 mAbs.

### ELISA

Various fusion proteins were coated to an ELISA plate (0.5ug/well). Blocking was performed with BSA 1%. 0.1ug/well of biotinylated HA-Ig was added followed by incubation with Streptavidin HRP (Jackson ImmunoResearch). Subsequently TMB solution is added and binding is measured by a spectrophotometer at an OD of 650nm.

## SUPPLEMENTARY MATERIAL FIGURE


