# Supplementary material for: The human 2B4 and NTB-A receptors bind the influenza viral hemagglutinin and co-stimulate NK cell cytotoxicity
Source: Oncotarget. 2016 Feb 22;7(11):13093–105. doi: 10.18632/oncotarget.7597 (PMC4914344; doi:10.18632/oncotarget.7597)
Supplement: Supplementary file 1 [file oncotarget-07-13093-s001.pdf]

The human 2B4 and NTB-A receptors bind the influenza viral hemagglutinin and co-stimulate NK cell cytotoxicity

Supplementary Materal

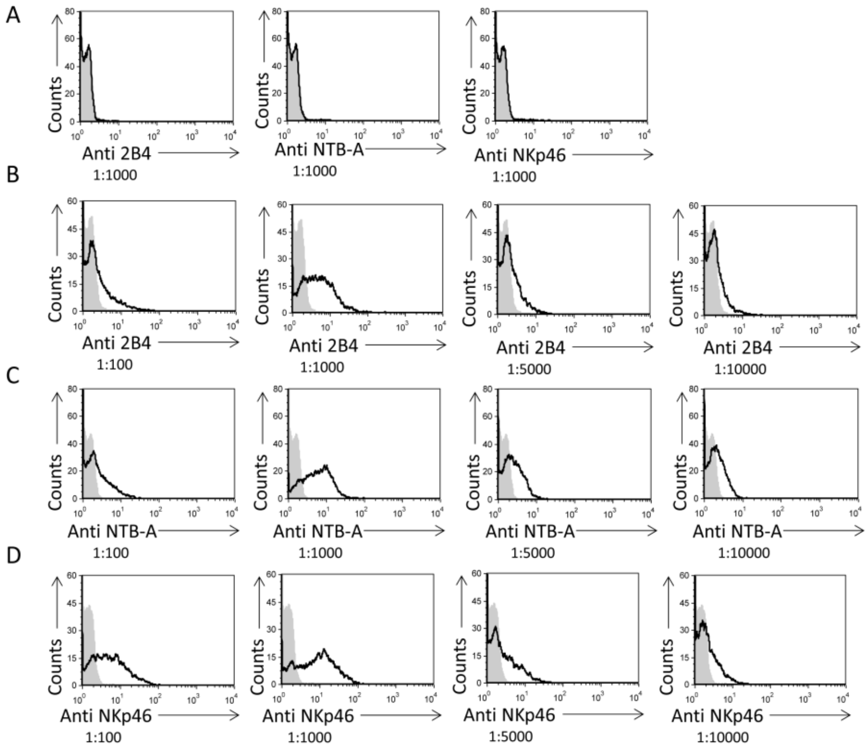

Figure S1: Specificity of 2B4, NTB-A and NKp46 specific sera

(A) BW parental cells that do not express 2B4, NTB-A or NKp46 were stained with the different sera (anti 2B4, anti NTB-A and anti NKp46). The black empty histogram represents the staining with the different sera and the filled histogram represents the staining of the BW cells with secondary antibodies only.

(B-D) Anti 2B4 (B), anti NTB-A (C) and anti NKp46 (D) polyclonal antibodies were used to stain BW cells expression 2B4 (B), NTB-A (C) or NKp46 (D). Various dilutions 1:100, 1:1000, 1:5000 and 1:10000 were tested. The black empty histogram represents the staining with the different sera and the filled histogram represents the staining of the BW cells with secondary antibodies only.
